# Construction of a Duck Intestinal Organoid Culture System: From Crypt Isolation to Medium Optimization

**DOI:** 10.3390/ani15213145

**Published:** 2025-10-29

**Authors:** Rui Tang, Xiang Luo, Li Zhang, Zhenhua Liang, Yan Wu, Jingbo Liu, Jinsong Pi, Hao Zhang

**Affiliations:** 1 Hubei Key Laboratory of Animal Embryo and Molecular Breeding, Institute of Animal Husbandry and Veterinary Sciences, Hubei Academy of Agricultural Sciences, No. 1 Nanhuyaoyuan, Nanhu Road, Wuhan 430064, China; q1207515167@163.com (R.T.); luoxiangcsq@163.com (X.L.); zl9588623@163.com (L.Z.); 13647253317@163.com (Z.L.); wuyanwh@163.com (Y.W.); pijinsong@hbaas.com (J.P.); 2 School of Life Science and Engineering, Southwest University of Science and Technology, Mianyang 621010, China; liuswust@163.com

**Keywords:** duck, crypts, intestinal organoids, culture system

## Abstract

**Simple Summary:**

Understanding how the intestine grows and functions is important for animal health and production. Organoids are miniature three-dimensional structures grown in the lab that can mimic real intestinal tissues. They serve as useful tools for studying gut development, disease mechanisms, treatment strategies, and how microbes interact with the host. While organoid culture systems for chickens are well developed, similar systems for ducks are still lacking. In this study, we established a culture method better suited to duck intestines by optimizing the isolation of intestinal stem cells and refining the growth conditions. We found that a solution called ethylene glycol tetraacetic acid was most effective for isolating duck intestinal crypts, and that suspension culture helped the organoids survive, grow, and develop better than other methods. We also discovered that adding natural factors such as serum, retinol, and retinoic acid significantly improved the formation and early differentiation of the organoids. This work establishes the first reproducible model of duck intestinal organoids, highlighting its potential as a foundation for further research and practical applications in poultry science.

**Abstract:**

Intestinal organoids possess self-organizing capacity and recapitulate essential features of intestinal architecture and function, making them powerful models for investigating development, disease mechanisms, pharmacological testing, and host–microbe interactions. Although standardized protocols for chicken intestinal organoids have been established, a defined culture system for ducks has not been available. In this study, we optimized crypt isolation procedures and culture medium composition to establish a reproducible system tailored to duck intestinal stem cells. Among various digestive solutions, ethylene glycol tetraacetic acid (EGTA) achieved the highest crypt isolation efficacy and organoid survival. Suspension culture resulted in better survival, proliferation, and differentiation of intestinal stem cells than air–liquid interface and embedding methods (*p* < 0.05). Immunofluorescence and real-time PCR indicated the presence of multiple epithelial lineages, including stem cells, Paneth cells, enterocytes, goblet cells, and enteroendocrine cells. Media supplemented with CHIR99021 and LDN193189 (CL) supported growth comparable to that of media with EGF, Noggin, and R-spondin 1 (ENR). Duckling serum and specific factors, such as SB203580 and retinol, further improved organoid formation and promoted differentiation. While long-term passaging and expansion remain technically challenging, this work provides the first duck intestinal organoid model and lays the foundation for future applications in avian intestinal research, including nutrition, disease modeling, and intervention strategies.

## 1. Introduction

China leads the world in terms of duck production, both for meat and eggs. The Chinese duck farming industry is gradually transitioning from traditional practices to scaled and modernized facilities. To optimize flock health and farming efficacy [1,2], researchers have increasingly studied duck intestinal nutrition, enteric diseases, and stress-induced intestinal mucosal damage [3]. To address persistent challenges in duck farming—including enteric infections, nutrient malabsorption, antibiotic overuse, and stress-induced intestinal damage—there is an urgent need for advanced in vitro models to study nutrient absorption mechanisms, host–microbiota interactions, and intestinal epithelial repair. Therefore, this study aims to establish a reliable and reproducible duck intestinal organoid culture system. Sato et al. [4] developed intestinal organoids using LGR5^+^ stem cells, providing a novel model for studying intestinal mucosal functions. Intestinal organoids can simulate the cellular composition and states of organs in vitro while maintaining stem cell pluripotency. These models are widely used in disease modeling [5,6,7], drug screening [8,9] and host–microbe interaction studies [10,11], making them powerful tools for research on development and disease [4,12].

Given the importance of intestinal organoids in in vitro studies, organoid culture systems for humans [13,14], mice [15], and chickens [16] have been gradually refined. Mammalian intestinal stem cells are typically isolated via collagenase [17,18] or EDTA [19,20], while EGTA is frequently used for chickens [21,22]. Isolated crypts are typically embedded in Matrigel and plated in the bottom of 12-well plates (embedding method) [23,24], cultured in a Transwell system using the ALI method [25], or suspended in ultralow attachment plates for suspension culture. However, a culture method for duck intestinal organoids has yet to be established.

Unlike traditional cell culture, which cannot support the 3D growth of isolated intestinal crypts, intestinal organoid culture media are specifically supplemented with three growth factors: epidermal growth factor (EGF), Noggin, and R-spondin 1 (collectively referred to as ENR). EGF promotes the growth of intestinal cells [26], whereas Noggin, a member of the transforming Growth Factor-β (TGF-β) superfamily, binds to BMP4 and BMP7 as a secreted inhibitor of bone morphogenetic proteins (BMP). This molecule acts in opposition to BMPs, coordinating Wnt signaling to activate and induce the proliferation of stem cells when bound to BMP [27]. R-spondin 1, a Wnt signaling activator, increases Wnt signaling to promote the proliferation and differentiation of intestinal stem cells while maintaining stemness [28]. However, the high cost of Noggin and R-spondin 1 significantly increases experimental costs, and EGF has been proven to be nonessential in organoid culture media [29]. As a result, alternative substances have been studied and applied in recent years. For instance, the biosynthetic Wnt activator CHIR99021 and BMP inhibitor LDN-193189 are used as substitutes for traditional ENR medium components. Additionally, the impact of small molecules on organoid culture has drawn increasing attention. Activin A modulates TGF-β signaling to influence the proliferation and differentiation of intestinal cells [30,31]. The combined use of the TGF-β receptor inhibitor SB431542 and the mitogen-activated protein kinase (MAPK) pathway inhibitor SB203580 was shown to regulate the proliferation and differentiation of intestinal organoids [32]. Inhibition of the ROCK signaling pathway is also known to support organoid formation and stem cell maintenance; ZINC00881524, a ROCK inhibitor, may hold potential in this regard, although its specific effects in intestinal organoids remain to be fully explored [33]. Moreover, vitamin A (VA) and its derivative retinoic acid (RA) play crucial roles in maintaining the proliferation, differentiation, and functionality of intestinal epithelial cells by affecting Wnt [34] and RA signaling pathways [35]. Although these small-molecule modulators have been applied in mammalian intestinal organoid cultures, their effects on the duck intestinal organoid culture system remain unclear.

In this study, we compared current methods for digesting and culturing duck intestinal organoids to identify the optimal isolation approach. Furthermore, various culture methods and media components were evaluated to establish an initial, yet reproducible, duck intestinal organoid system that may serve as a platform for future optimization. This foundation is expected to facilitate future studies in avian intestinal health, nutrition, and disease.

## 2. Materials and Methods

### 2.1. Sample Collection

After twenty-six to twenty-seven days of incubation, duck embryos were sterilized with 75% ethanol. All ducks were subsequently euthanized via carotid artery exsanguination after being anesthetized via intravenous injection of dexmedetomidine hydrochloride (8 mg/kg, X-1251, SIGMA, Kawasaki, Japan). Under sterile conditions, the small intestine of the duck embryos was isolated. The small intestine was immersed in PBS containing 3% penicillin/streptomycin (15140148, Gibco, Grand Island, NY, USA). After three washes with PBS, the mesentery was removed, and the intestines were opened longitudinally. The intestinal contents were thoroughly rinsed, and the tissue was cut into segments approximately 1 cm in length.

### 2.2. Screening Digestive Solutions for Crypt Stem Cell Isolation

Duck intestinal segments were transferred into 15 mL centrifuge tubes and digested via three different solutions: (1) 2.5 mM EGTA solution (HY-D0861, MedChemExpress, Monmouth Junction, NJ, USA): The segments were initially digested with 10 mL of 2.5 mM EGTA solution on a shaker at 120 rpm for 15 min. The solution was then replaced with 10 mL of a fresh 2.5 mM EGTA solution, and the mixture was digested for 45 min. The entire process was carried out at 4 °C. (2) 30 mM EDTA solution (HY-Y0682, MedChemExpress): The segments were digested with 30 mM EDTA solution on a shaker at 120 rpm for 60 min, and the entire process was performed at 4 °C. (3) Type I collagenase (1%): The segments were digested with 1% type I collagenase (17018029, Gibco, Waltham, MA, USA) at 37 °C for 60 min, with gentle shaking of the centrifuge tube every 10~20 min. After digestion, the intestinal segments were transferred to DMEM/F12 medium and gently pipetted for approximately 10 min. The mixture was filtered through a 100 μm cell strainer and centrifuged, and the supernatant was discarded. The pellet was resuspended in DMEM/F12 medium, and 10 μL of the suspension was placed onto a glass slide for microscopic observation and crypt density calculation. The crypts were then transferred to ENR medium and cultured in an incubator at 37 °C with 5% CO_2_. During the culture process, the morphology of the organoids was observed and recorded every two days via a microscope, with fresh medium replaced regularly.

### 2.3. Preparation of Intestinal Tissue Paraffin Sections and H&E Staining

The digestive products obtained from the three different solutions were collected and preserved in 4% paraformaldehyde. The samples were then embedded in paraffin and sectioned horizontally into 4 μm thick slices. After deparaffinization and rehydration, the intestinal sections were stained with hematoxylin and eosin. The stained sections were observed under a Nikon E200 microscope (Nikon, Tokyo, Japan) to examine the morphology of the intestinal mucosa. By comparing the images, changes in the duodenal crypts before and after intestinal digestion were evaluated.

### 2.4. Screening of Duck Intestinal Organoid Culture Methods

**Figure 1:**
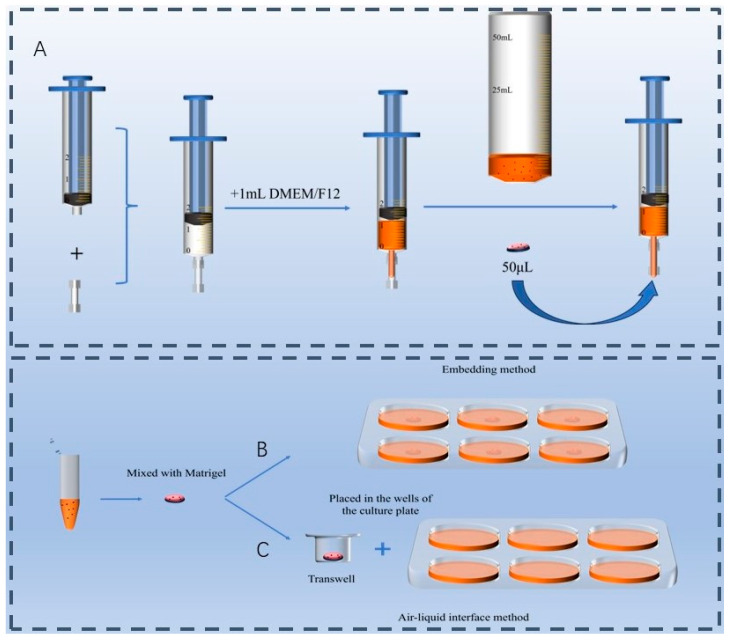
Culture devices. (**A**) Custom-made suspension culture device using a syringe. (**B**) Embedding method. (**C**) Air–liquid interface culture method.

Three culture methods were tested: the suspension method, the embedding method and the ALI culture method. (1) Suspension culture method: For this method, intestinal crypts were not mixed with Matrigel. Instead, 1 mL of ENR medium was added to a syringe, and 50 μL of intestinal crypt suspension was introduced to the tip of the syringe by a micropipette (Figure 1A). (2) Embedding method (Figure 1B): Intestinal crypts were mixed with Matrigel in equal volumes and placed at the center of a 6-well plate. The plate was incubated at 37 °C in a cell culture incubator for 30 min to solidify the gel. Subsequently, 2 mL of ENR medium was added to each well to continue the culture. (3) ALI method (Figure 1C): Intestinal crypts were mixed with Matrigel in equal volumes and placed at the center of the upper chamber of a Transwell plate (140640, Gibco). The Transwell was incubated at 37 °C in a cell culture incubator for 30 min to allow the gel to solidify. Afterward, 250 μL of ENR medium was added to the upper chamber, and 750 μL was added to the lower chamber for continued culture. The medium in the upper chamber was removed during the next medium change to establish an air–liquid interface. Approximately 400 intestinal crypts were cultured via each method and placed in a 37 °C, 5% CO_2_ incubator. Organoid morphology was observed and photographed every two days under a microscope, with fresh culture medium being replaced at the same intervals. Quantitative analysis of organoid number and diameter was performed via ImageJ 1.5 digital processing software.

### 2.5. Screening and Optimization of Culture Media

After the crypt suspension was obtained, the following three types of culture media were used for cultivation: (1) ENR medium: This medium contained 1 mg/mL epidermal growth factor (EGF, HY-P7109, MedChemExpress), 100 μg/mL Noggin (HY-P700143AF, MedChemExpress), and 500 μg/mL R-spondin 1 (HY-P7114, MedChemExpress). (2) Organoid growth medium (OGM): This was a commercial medium (06005, STEMCELL Technologies Inc., Vancouver, BC, Canada). (3) CL medium: This medium contained 15 μM CHIR-99021 (HY-10182, MedChemExpress) and 0.3 μM LDN-193189 (HY-12071, MedChemExpress). All three media were supplemented with 1% penicillin/streptomycin (15140148, Gibco), 2 mM GlutaMAX (35050068, Gibco), and 10 mM HEPES (15630080, Thermo Fisher Scientific, Waltham, MA, USA). Additionally, for optimization of the culture media, the following reagents were tested: 100 ng/mL activin A (HY-P70311, MedChemExpress), 0.5 μM SB431542 (HY-10431, MedChemExpress), 10 μM SB203580 (HY-10256, MedChemExpress), 25 mM ZINCE00881524 (HY-101244, MedChemExpress), different concentrations of VA/RA (16174, Merck, St. Louis, MO, USA), and serum collected from the blood of ducklings, adult laying ducks, and meat ducks. The blood was centrifuged at 4000 rpm, and the supernatant was filtered through a 0.22 μm filter. An appropriate amount of medium was added to each well, and the cultures were maintained at 37 °C with 5% CO_2_ in a cell culture incubator. The medium was replaced every two days, and organoid growth was documented on days 0, 2, and 4 of culture.

### 2.6. Cell Viability Assay

The viability of the intestinal organoids was assessed via the CellTiter-Glo^®^ 3D Reagent Kit (G9681, Promega Corporation, Madison, WI, USA). First, the cultured intestinal organoids were transferred to a 1.5 mL centrifuge tube. After centrifugation to remove the culture medium, the organoids were resuspended in PBS. Next, an equal volume of pre-equilibrated CellTiter-Glo^®^ 3D Reagent (1:1 ratio) was added to the suspension. The mixture was thoroughly mixed and aliquoted into a 384-well plate, followed by shaking for 5 min. The plate was then incubated at room temperature for 25 min, and luminescence was measured via a microplate reader.

### 2.7. RNA Extraction and Real-Time Quantitative PCR

The cultured intestinal organoids were transferred to a 1.5 mL centrifuge tube, and the supernatant was removed by centrifugation. Total RNA was isolated from the organoids using the TRIzol method (Invitrogen, Carlsbad, CA, USA), and DNA contamination was removed from the samples using RQ1 DNase (Promega). RNA concentration was measured using a NanoDrop™ 2000 spectrophotometer (Thermo Fisher Scientific, Waltham, MA, USA), and RNA integrity was assessed using the Agilent Bioanalyzer 2100 system (Agilent Technologies, Santa Clara, CA, USA). Approximately 1 μg of total RNA was used for cDNA synthesis using the SuperScript™ IV Reverse Transcriptase kit (Invitrogen). Real-time quantitative PCR was performed on a LightCycler 96 system (Roche, Life Science, Munich, Germany) using SYBR Green Master Mix (Vazyme, Nanjing, China). Each 10 μL reaction mixture contained 5 μL of SYBR Green Master Mix, 0.5 μL of forward primer, 0.5 μL of reverse primer, 1.5 μL of cDNA, and 2.5 μL RNase-free water. Forward and reverse primers were used at a final concentration of 0.5 μM, prepared from 10 μM stock solutions. The RT-qPCR amplification conditions were as follows: 95 °C for 40 s for initial denaturation, followed by 40 cycles of 95 °C for 10 s and 60 °C for 30 s for annealing. Primers for different cell types were designed using the NCBI database, including the intestinal stem cell marker leucine-rich repeat-containing G-protein-coupled receptor 5 (*LGR5*), the goblet cell marker Mucin 2 (*MUC2*), the Paneth cell marker Lysozyme (*LYZ*), the enteroendocrine cell marker Chromogranin A (*CHGA*), and the enterocyte marker sucrase-isomaltase (*SI*), as shown in Table 1. Relative mRNA expression levels were calculated using the 2^−ΔΔCt^ method.

### 2.8. Immunofluorescence Staining for Intestinal Organoid Markers

After recovering the organoids from the syringe, they were transferred to a 1.5 mL centrifuge tube and centrifuged to remove the supernatant. The organoids were resuspended in PBS and transferred to a 24-well plate, allowing them to settle at the bottom of the wells. An appropriate amount of 4% paraformaldehyde (Invitrogen) was added, and the organoids were fixed at room temperature for 30 min. Following fixation, the organoids were washed with PBS and then blocked with fetal bovine serum (Invitrogen) for 30 min at room temperature. After blocking, the organoids were washed again with PBS, and the primary antibody (LGR5, 1:250, DF2816, Affinity Biosciences, Cincinnati, OH, USA) was added. The samples were incubated overnight at 4 °C. After removing the primary antibody and washing with PBS, the secondary antibody (FITC conjugate) was added and incubated in the dark for 30 min. The secondary antibody was then discarded, and the organoids were washed with PBS. Subsequently, the organoids were treated with 0.5% Triton-X-100 (Invitrogen) for 5 min. Next, 200–500 μL of prepared rhodamine-labeled phalloidin working solution (Invitrogen) was added to cover the organoids, and the samples were incubated at room temperature in the dark for 30 min. After discarding the working solution and washing with PBS, DAPI staining solution (Vector Laboratories, Burlingame, CA, USA) was added to completely cover the cells, and they were incubated in the dark at room temperature for 3–5 min. The staining solution was discarded, and the organoids were washed with PBS. Finally, the stained organoids were observed under a fluorescence microscope.

### 2.9. Statistical Analysis

SPSS statistical software (version 26.0, IBM Corp., Armonk, NY, USA) was used to analyze all the data from these experiments, while GraphPad Prism software (version 8.0, GraphPad Software, San Diego, CA, USA) was used to create experimental graphs. Differences between two groups were identified via t-test, while differences among multiple groups were assessed using one-way analysis of variance (ANOVA). The data are presented as the means ± standard errors of the means (SEMs) from three independent experiments, with *p*-values less than 0.05 considered statistically significant.

## 3. Results

### 3.1. Effects of Digestive Solutions on the Isolation and Culture of Duck Intestinal Crypts

**Figure 2:**
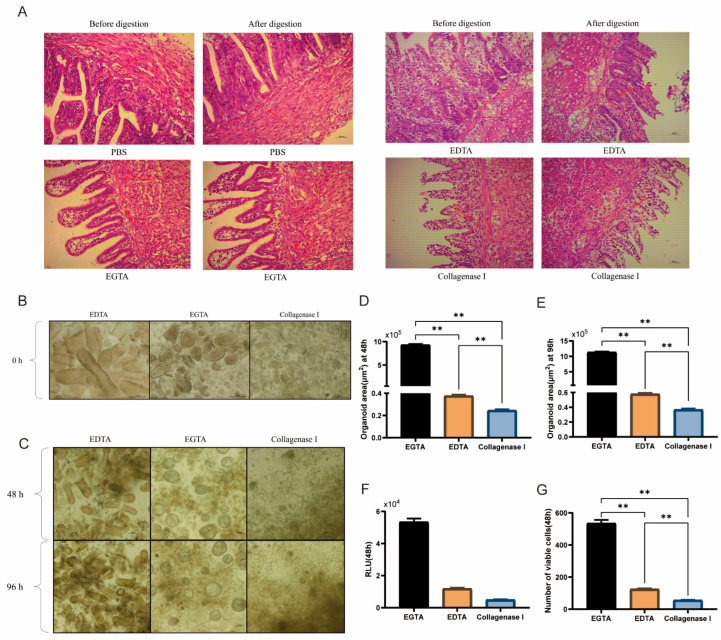
Effects of digestive solutions on the isolation and culture of duck intestinal crypts. (**A**) H&E staining: Crypt isolation before and after digestion with PBS, EDTA, EGTA, and Type I collagenase; arrows in (**A**) indicate the positions of intestinal crypts before and after digestion; scale bar = 100 μm. (**B**) Microscopic observation of cell suspensions after treatment with three digestive fluids (prior to plating); scale bar = 100 μm. (**C**) Microscopic observation of organoid growth after 48 h and 96 h of treatment with EGTA, EDTA, and Type I collagenase; scale bar = 100 μm. (**D**,**E**) Organoid area calculation 48 h after treatment with three digestive fluids (*n* = 3). (**F**) Enzyme activity assay results from microplate reader. (**G**) Cell viability assay 48 h after treatment with three digestive fluids (*n* = 3). Values are presented as the means ± SE. ** means *p* < 0.01.

As shown in Figure 2A,B, EDTA primarily affects the villi rather than the crypts. Type I collagenase has a minimal effect on crypt digestion but effectively dissociates duck intestinal epithelial cells. In contrast, EGTA yields the most structurally intact and abundant crypts in the digestive suspension. After 48 and 96 h of culture, the organoid surface area in the EGTA-treated group was significantly higher than that in the EDTA- and Type I collagenase-treated groups (*p* < 0.01, Figure 2D,E). Moreover, at 48 h of culture, the number of viable cells in the EGTA-treated group was significantly higher (*p* < 0.01, Figure 2F,G) than in the other two groups. Thus, EGTA not only demonstrates higher efficiency in crypt isolation but also has less impact on subsequent crypt culture. In conclusion, EGTA is better suited as a digestive solution for isolating duck intestinal crypts.

### 3.2. Effects of Culture Methods on Duck Intestinal Organoid Development

**Figure 3:**
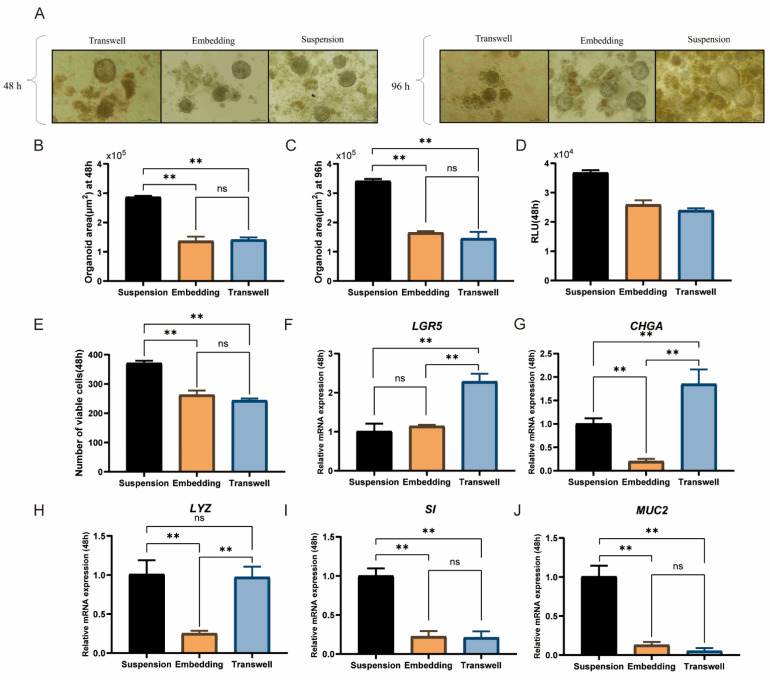
Effects of culture methods on duck intestinal organoid development. (**A**) Microscopic observation of intestinal organoids cultured for 48 h and 96 h using air–liquid interface, embedding, and suspension methods; scale bar = 100 μm. Organoid area after 48 h (**B**) and 96 h (**C**) of culture using the three methods (*n* = 3). (**D**) Enzyme activity assay results from microplate reader. (**E**) Cell viability assay after 48 h of culture using the three methods (*n* = 3). Relative mRNA expression levels of stem cell marker (**F**) leucine-rich repeat-containing G protein-coupled receptor 5 (*LGR5*), endocrine cell marker (**G**) chromogranin A (*CHGA*), Paneth cell marker (**H**) lysozyme (*LYZ*), enterocyte marker (**I**) sucrase-isomaltase (*SI*), and goblet cell marker (**J**) mucin 2 (*MUC2*) in different treatment groups after 48 h of culture (*n* = 3). Values are presented as the means ± SE. ** means *p* < 0.01, ns indicates no significant difference.

As shown in Figure 3A, intestinal organoids cultured via the ALI and embedding methods grew significantly larger at 96 h than they did at 48 h, with diameters ranging mostly from 100 μm to 130 μm. However, the density and size of the organoids cultured via the suspension method with the same seeding density were significantly greater than those of the other two groups. Their diameters ranged from 100 μm to 200 μm, and differentiation was observed. After 48 and 96 h of culture, the organoid areas in the suspension group were 2.8 × 10^5^ μm^2^ and 3.3 × 10^5^ μm^2^, respectively, both of which were significantly greater than those in the ALI and embedding groups (*p* < 0.01, Figure 3B,C). Additionally, cell viability tests at 48 h indicated that the suspension group had significantly greater viability than the other two groups did (*p* < 0.01, Figure 3D,E).

Compared with the embedding method, the suspension culture method significantly increased the gene expression levels of *LYZ*, a marker of Paneth cells; *SI,* a marker of enterocytes; and *MUC2*, a marker of goblet cells (*p* < 0.01, Figure 3H–J). In contrast, the ALI method resulted in significantly greater expression levels of *LGR5* and *CHGA*, which are markers associated with intestinal stem cell stemness, than the suspension and embedding methods did (*p* < 0.01, Figure 3F,G), indicating its effectiveness in maintaining intestinal stem cell stemness. However, the ALI group presented a lower cell density. In comparison, the suspension culture group, despite lower *LGR5* expression, better supported intestinal stem cell differentiation and maintained a higher cell density, making it more suitable for subsequent experiments. In conclusion, the suspension culture method is better suited for cultivating duck intestinal organoids.

### 3.3. Effects of Different Culture Media on Duck Intestinal Organoid Growth and Identification

**Figure 4:**
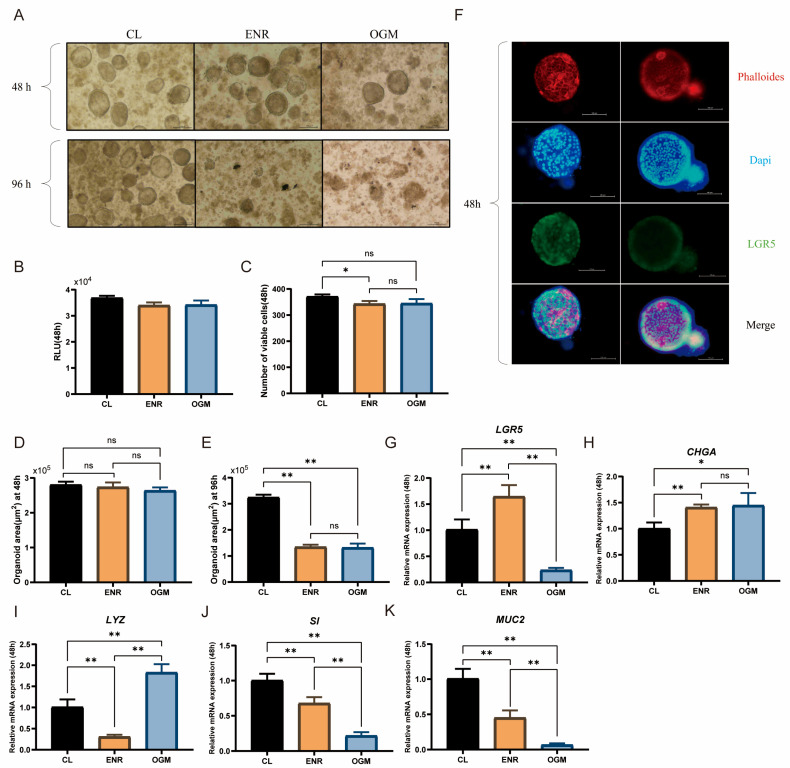
Effects of different culture media on duck intestinal organoid growth and identification. (**A**) Growth of intestinal organoids cultured in ENR, CL, and OGM media for 48 h and 96 h; scale bar = 100 μm. (**B**,**C**) Cell viability results after 48 h of culture in CL, ENR, and OGM media (*n* = 3). (**D**) Organoid area after 48 h of culture in CL, ENR, and OGM media (*n* = 3). (**E**) Organoid area after 96 h of culture in CL, ENR, and OGM media (*n* = 3). (**F**) Representative immunofluorescence images of duck intestinal organoids, showing cytoskeleton stained in red (phalloidin), nuclei stained in blue (DAPI), intestinal stem cells stained in green (*LGR5* antibody), and merged image; scale bar = 100 μm. Relative mRNA expression levels of stem cell marker (**G**) leucine-rich repeat-containing G protein-coupled receptor 5 (*LGR5*), endocrine cell marker (**H**) chromogranin A (*CHGA*), Paneth cell marker (**I**) lysozyme (*LYZ*), enterocyte marker (**J**) sucrase-isomaltase (*SI*), and goblet cell marker (**K**) mucin 2 (*MUC2*) in different treatment groups after 48 h of culture (*n* = 3). Values are presented as the means ± SE. * indicates *p* < 0.05, ** means *p* < 0.01, ns indicates no significant difference.

After 48 h of culture, the viability of the duck intestinal organoids in the CL group was significantly greater than that in the ENR group (*p* < 0.05, Figure 4B,C), with an area of approximately 2.7 × 10^5^ μm^2^, which was not significantly different from that in the ENR and OGM groups (Figure 4D). Quantitative analysis further revealed that the CL group outperformed the ENR and OGM groups in promoting the proliferation of Paneth cells, enterocytes, and goblet cells (*p* < 0.05, Figure 4I–K). However, the expression levels of *LGR5* and *CHGA* in the CL group were significantly lower than those in the ENR group (*p* < 0.05, Figure 4G,H). After 96 h of culture, the organoid diameter in the CL group increased from 100 μm to 150 μm (Figure 4A), and a cavity-like cellular morphology was observed. The area expanded to approximately 3.2 × 10^5^ μm^2^, which was significantly greater than that of the ENR and OGM groups (*p* < 0.01, Figure 4E), whereas the organoids in the latter two groups exhibited fragmentation and death. In summary, although CL medium has slight limitations in maintaining intestinal stem cell stemness, it effectively promotes intestinal stem cell differentiation and is suitable for long-term experiments.

The results of immunofluorescence staining also demonstrated the expression of LGR5 in the intestinal organoids (Figure 4F), confirming the similarity between the intestinal organoids derived from duck intestinal crypt stem cells cultured in self-prepared CL medium and the in vivo epithelial system.

### 3.4. Duck Serum Supplementation Significantly Increases the Proliferation and Differentiation of Duck Intestinal Organoids

**Figure 5:**
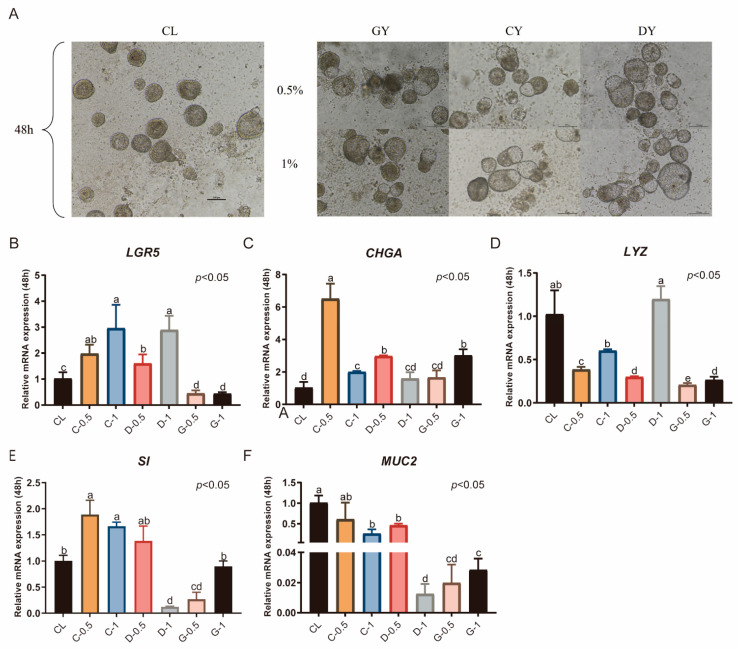
Duck serum supplementation significantly enhances the proliferation and differentiation of duck intestinal organoids. (**A**) Microscopic observation of organoids cultured for 48 h after serum addition; scale bar = 100 μm. Relative mRNA expression levels of stem cell marker (**B)** leucine-rich repeat-containing G protein-coupled receptor 5 (*LGR5*), endocrine cell marker (**C**) chromogranin A (*CHGA*), Paneth cell marker (**D**) lysozyme (*LYZ*), enterocyte marker (**E**) sucrase-isomaltase (*SI*), and goblet cell marker (**F**) mucin 2 (*MUC2*) in different treatment groups after 48 h of culture (*n* = 3). Values are presented as the means ± SE. The means with different superscript letters indicate statistically significant differences (*p* < 0.05). Abbreviations: CL = supplemented with CHIR99021 and LDN193189; GY = Serum extracted from male ducks; DY = Serum extracted from laying ducks; CY = Serum extracted from ducklings; C-0.5 = Containing 0.5% duckling serum; C-1 = Containing 1% duckling serum; D-0.5 = Containing 0.5% adult laying duck serum; D-1 = containing 1% adult laying duck serum. G-0.5 = Containing 0.5% adult drake serum; and G-1 = Containing 0.5% adult drake serum.

In this study, serum from male ducks (GY), laying ducks (DY), and ducklings (CY) was added to CL medium lacking N_2_ or B_27_, and the outcomes were compared with those in CL medium supplemented with N_2_ or B_27_. Overall, organoid diameters across treatments remained around 100 μm. Under the GY condition, 0.5% serum was sufficient for a subset of organoids to reach 100 μm, and increasing the concentration to 1% had no appreciable effect on diameter. With CY, diameters were comparable to those under GY and remained largely stable as the concentration increased. Under DY, most organoids likewise maintained diameters near 100 μm. In the 0.5% DY group, the number of buds was relatively low, with most organoid diameters within 100 μm. At a concentration of 1%, the diameter increased significantly (Figure 5A). Compared with the CL medium supplemented with serum, 0.5% CY significantly increased the expression levels of *LGR5*, *CHGA*, and *SI* (*p* < 0.05, Figure 5B,C,E). At a concentration of 1%, the expression of *LGR5* and *CHGA* was significantly elevated (*p* < 0.01, Figure 5B,C), whereas the expression of *LYZ* was reduced (*p* < 0.05, Figure 5D). The expression level of *CHGA* in the 0.5% CY group was much greater than that in the 1% CY group (*p* < 0.05, Figure 5C), with no significant differences observed in the expression of *SI* and *MUC2*. *LYZ* expression was significantly greater in the 1% DY group than in the CY group (*p* < 0.05, Figure 5D), whereas the *SI* and *MUC2* expression levels were significantly lower (*p* < 0.05, Figure 5E,F). In the GY groups, the expression levels of all five marker genes were lower than those in the 0.5% CY group.

### 3.5. Impact of Activin a and ZINC00881524 on the Culture of Duck Intestinal Organoids

**Figure 6:**
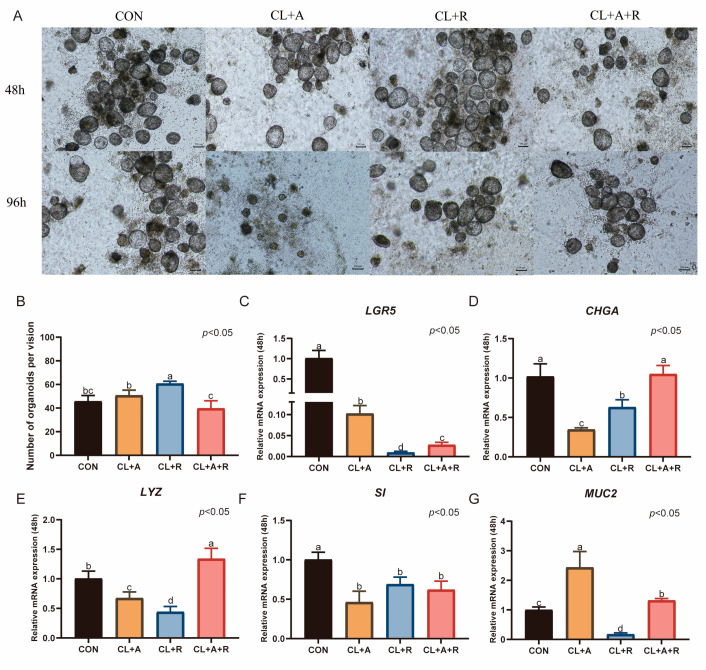
Impact of activin a and ZINC00881524 on the culture of duck intestinal organoids. (**A**) Growth of duck intestinal organoids in different treatment groups at 48 h and 96 h; scale bar = 100 μm. (**B**) Number of intestinal organoids in different treatment groups, with organoids counted in at least three microscopic fields of view. Relative mRNA expression levels of stem cell marker (**C**) leucine-rich repeat-containing G protein-coupled receptor 5 (*LGR5*), endocrine cell marker (**D**) chromogranin A (*CHGA*), Paneth cell marker (**E**) lysozyme (*LYZ*), enterocyte marker (**F**) sucrase-isomaltase (*SI*), and goblet cell marker (**G**) mucin 2 (*MUC2*) in different treatment groups after 48 h of culture (*n* = 3). Values are presented as the means ± SE. The means with different superscript letters indicate statistically significant differences (*p* < 0.05). Abbreviations: CON = Control group; CL + A = supplemented with CHIR99021, LDN193189, and Activin A; CL + R = supplemented with CHIR99021, LDN193189, and ZINC00881524; and CL + A + R = supplemented with CHIR99021, LDN193189, Activin A, and ZINC00881524.

Treatment with ZINC00881524 alone for 48 h significantly increased the number of duck intestinal organoids (*p* < 0.05, Figure 6B). When activin A was used alone or in combination with ZINC00881524, the number of organoids was similar to that in the CON group, which was cultured in CL medium supplemented with 0.5% duckling serum (*p* > 0.05, Figure 6B). Nevertheless, after 96 h, the number of organoids decreased. Treatment with activin A alone significantly increased the expression of *MUC2* after 48 h of culture (*p* < 0.05, Figure 6G). In contrast, this treatment reduced the expression of *LGR5*, *CHGA*, *LYZ*, and *SI* (*p* < 0.05). Compared with activin A treatment alone, ZINC00881524 treatment alone significantly elevated *CHGA* expression (*p* < 0.05, Figure 6D). However, the expression levels of *LGR5*, *CHGA*, *LYZ*, *SI*, and *MUC2* were lower than those in the CON group (*p* < 0.05, Figure 6C–G). The combined treatment with activin A and ZINC00881524 upregulated *LYZ* expression (*p* < 0.05, Figure 6E) and maintained *CHGA* expression. However, this treatment significantly downregulated the expression of *LGR5*, *SI*, and *MUC2* (*p* < 0.05).

### 3.6. Effects of SB431542 and SB203580 on the Culture of Duck Intestinal Organoids

**Figure 7:**
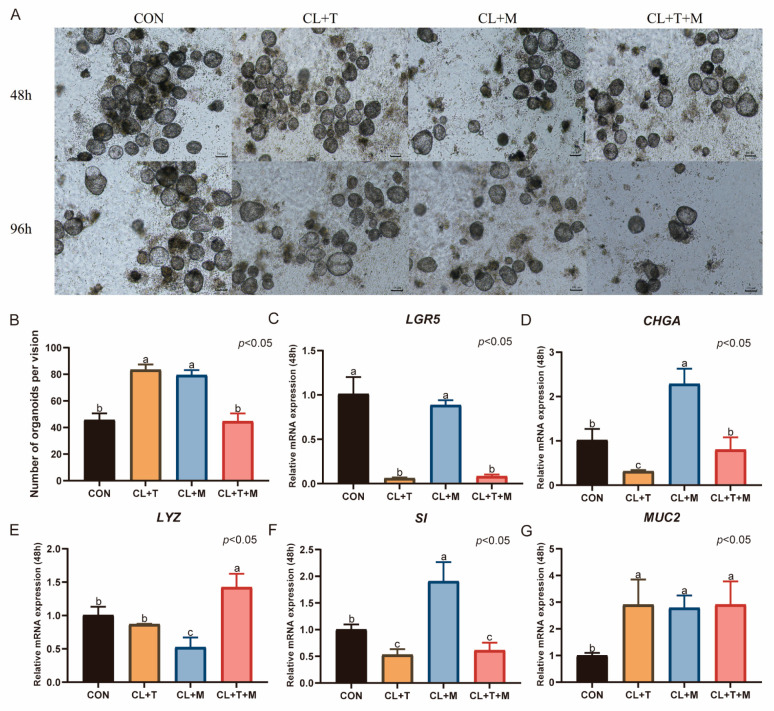
Effects of SB431542 and SB203580 on the culture of duck intestinal organoids. (**A**) Growth of duck intestinal organoids in different treatment groups at 48 h and 96 h; scale bar = 100 μm. (**B**) Number of intestinal organoids in different treatment groups, with organoids counted in at least three microscopic fields of view. Relative mRNA expression levels of stem cell marker (**C**) leucine-rich repeat-containing G protein-coupled receptor 5 (*LGR5*), endocrine cell marker (**D**) chromogranin A (*CHGA*), Paneth cell marker (**E**) lysozyme (*LYZ*), enterocyte marker (**F**) sucrase-isomaltase (*SI*), and goblet cell marker (**G**) mucin 2 (*MUC2*) in different treatment groups after 48 h of culture (*n* = 3). Values are presented as the means ± SE. The means with different superscript letters indicate statistically significant differences (*p* < 0.05). Abbreviations: CON = Control group; CL + T = supplemented with CHIR99021, LDN193189, and SB431542; CL + M = supplemented with CHIR99021, LDN193189, and SB203580; and CL + T + M = supplemented with CHIR99021, LDN193189, SB431542, and SB203580.

After 48 h of organoid culture with the addition of SB431542, the number of organoids significantly increased (*p* < 0.05, Figure 7B). However, the number decreased after 96 h. Treatment with SB203580 alone also significantly increased the number of organoids (*p* < 0.05, Figure 7B), but a similar decrease was observed after 96 h. When SB431542 and SB203580 were used together, the number of organoids was comparable to that in the CON group, which was cultured in CL medium supplemented with 0.5% duckling serum, activin A, and ZINC00881524.

Compared with the CON group, the addition of SB431542 significantly increased the expression level of *MUC2* after 48 h of organoid culture (*p* < 0.05, Figure 7G). However, this treatment significantly downregulated the mRNA expression of *LGR5*, *CHGA*, and *SI* (*p* < 0.05, Figure 7C,D,F), with no effect on *LYZ* expression. Treatment with SB203580 alone significantly increased the expression of *CHGA*, *SI*, and *MUC2* (*p* < 0.05, Figure 7D,F,G), reduced the expression of *LYZ* (*p* < 0.05, Figure 7E), and had no effect on the mRNA expression of *LGR5*. The combined use of SB431542 and SB203580 increased the mRNA expression of *LYZ* and *MUC2* (*p* < 0.05, Figure 7E,G), decreased the mRNA expression of *LGR5* and *SI* (*p* < 0.05), and had no effect on *CHGA* expression.

### 3.7. Effects of VA and RA on the Culture of Duck Intestinal Organoids

**Figure 8:**
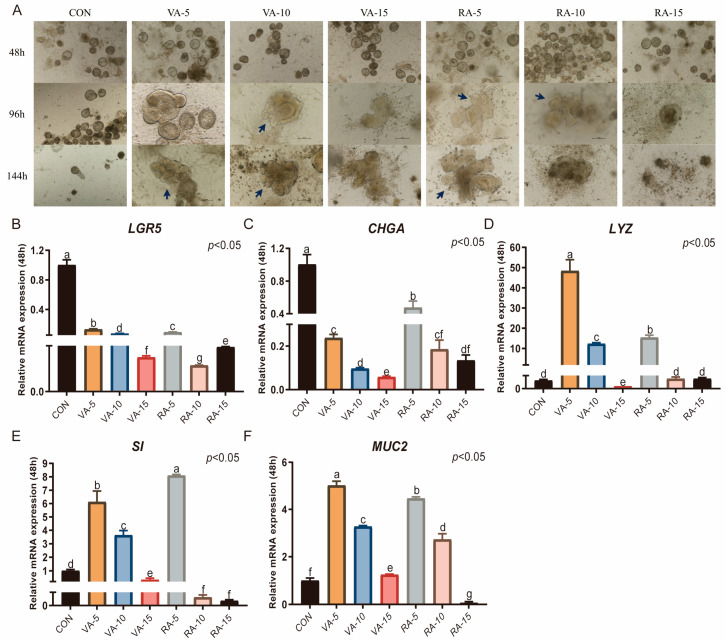
Effects of VA and RA on the culture of duck intestinal organoids. (**A**) Growth of duck intestinal crypt stem cells with different concentrations of VA and RA; arrows indicate the budding regions of organoids; scale bar = 100 μm. (**B**) Leucine-rich repeat-containing G protein-coupled receptor 5 (*LGR5*), endocrine cell marker (**C**) chromogranin A (*CHGA*), Paneth cell marker (**D**) lysozyme (*LYZ*), enterocyte marker (**E**) sucrase-isomaltase (*SI*), and goblet cell marker (**F**) mucin 2 (*MUC2*) in different treatment groups after 96 h of culture (*n* = 3). Values are presented as the means ± SE. The means with different superscript letters indicate statistically significant differences (*p* < 0.05). Abbreviations: CON = Control group; VA-5 = CL medium containing 5 IU of Vitamin A; VA-10 = CL medium containing 10 IU of Vitamin A; VA-15 = CL medium containing 15 IU of Vitamin A; RA-5 = CL medium containing 5 IU of Retinoic Acid; RA-10 = CL medium containing 10 IU of Retinoic Acid; and RA-15 = CL medium containing 15 IU of Retinoic Acid.

When different concentrations of VA and RA were added, budding and differentiation of duck intestinal crypt stem cells were observed after 96 h of culture at concentrations of 5 IU and 10 IU. Organoids displaying differentiated structures persisted for up to 144 h, extending the culture duration. However, when the concentration was increased to 15 IU, organoids fragmented within 96 h (Figure 8A). Therefore, the concentration of VA or RA should be kept below 15 IU. Compared with those in the CON group, which was cultured in CL medium supplemented with 0.5% duckling serum, activin A, ZINC00881524, and SB203580, the mRNA expression levels of the intestinal stem cell marker *LGR5* and the enteroendocrine cell marker *CHGA* significantly decreased after 96 h of culture (*p* < 0.05, Figure 8B,C). The expression of the other three markers increased to varying degrees. Specifically, at VA concentrations of 5 IU and 10 IU and an RA concentration of 5 IU, the expression levels of *LYZ*, *SI*, and *MUC2* significantly increased (*p* < 0.05, Figure 8D–F). In the VA-15 group and RA-10 group, only goblet cells were positively affected, with a significant increase in *MUC2* expression.

## 4. Discussion

In recent years, animal intestinal crypts cultured in vitro have become a reliable model for studying intestinal development, disease model construction, and drug screening. This technology has been widely applied and has achieved major progress in animals such as mice, pigs, cattle and chicken. However, research on duck intestinal organoids remains relatively scarce, leading to a lag in studies related to duck intestinal biology. Although chickens and ducks both belong to the avian class, their intestines differ significantly in developmental timing, physiological structure, barrier formation mechanisms, and metabolic demands [36,37]. Therefore, it is necessary to establish species-specific organoid culture systems. The intestinal development of chickens occurs earlier, with stable formation of tight junctions and microvilli, which facilitates early carbohydrate absorption. In contrast, ducks exhibit rapid intestinal structural changes after hatching and more active lipid metabolism, requiring different culture conditions [38]. Thus, establishing a duck-specific intestinal organoid model not only enables a more accurate simulation of its in vivo environment but also provides an important platform for studying intestinal development and disease mechanisms. In this study, crypt stem cells were isolated from embryonic duck intestines, and a duck intestinal organoid culture system was successfully established. Within this system, the key feature of crypt–villus-like structures was observed [39]. Additionally, the presence of intestinal stem cells, enteroendocrine cells, intestinal epithelial cells, goblet cells, and Paneth cells was successfully identified, reflecting the differentiation and maturation of intestinal cells [40]. However, their functional and structural features may not yet fully reflect those of mature intestinal tissue, suggesting that further optimization will be needed. Taken together, these results demonstrate the establishment of a reproducible duck intestinal organoid model that provides a practical basis for future refinement and application.

In the construction of an intestinal organoid culture system, isolation of crypts with self-organization from duck intestinal tissue is a critical step. Research has shown that crypt stem cells can be successfully isolated from the ileum of cats, cows, horses, pigs, and sheep via collagenase [17]. Organoid structures have been observed in ileal organoids from horses and cows. However, cat ileal organoids tend to undergo large-scale cell death during culture, and while pig and sheep ileal organoids can increase in size, they fail to form typical intestinal-like structures. When EDTA was used to isolate crypt stem cells from the jejunum and ileum of pigs, intestinal-like structures appeared on day 7. When EGTA was used to isolate crypt stem cells from chicken embryos, cavities formed on day 3 and fused into irregular spherical networks under the traction of myofibroblasts [21]. The characteristics of different species or intestinal regions determine the reagents and methods required for crypt isolation [41]. The H&E staining results in this study demonstrated that all three digestive solutions promoted the separation of crypts from the base of the villi. However, microscopic examination of the suspensions after digestion revealed that EDTA and type I collagenase were less effective at isolating duck intestinal crypt stem cells. In contrast, digestion with EGTA yielded more intact duck intestinal crypts, resulting in increased organoid diameter and survival rates during subsequent culture. Therefore, EGTA may be more suitable for isolating intestinal crypt stem cells from poultry.

The cultivation of most intestinal organoids relies on Matrigel, which provides an adhesion substrate for stem cells and facilitates intercellular signaling [42]. However, Matrigel has several drawbacks, including its undefined composition, lack of reproducibility and adjustability, and difficulty in handling during the culture process [43]. High ECM concentrations (≥20%) have been reported to lead to complete polymerization, forming a solid matrix layer that resembles dome embedding conditions. Under such conditions, the use of conventional culture plates may result in organoid adhesion and the loss of typical 3D structures [44]. The unique structure of the upper and lower chambers of Transwell membranes better simulates intestinal physiological processes, promoting the differentiation and maturation of intestinal organoids [45]. However, certain antibodies may nonspecifically bind to the Transwell membrane, affecting cell imaging [46]. Suspension culture has also been widely used for the cultivation of kidney [47] and pancreatic organoids [48]. While some iPSC-derived organoid models have demonstrated the capacity to spontaneously generate basement membrane structures [49], this may not directly apply to ASC-derived organoids due to cellular composition differences. Compared with Matrigel, suspension culture can increase organoid yield three- to fourfold and is thus considered a simpler, more effective method that is better suited for clinical applications. Similarly, in this study, the diameter and number of organoids obtained through suspension culture were significantly greater than those obtained via ALI culture or embedding methods. Although suspension culture reduced LGR5 expression, it significantly increased the expression of *MUC2* and *SI*, indicating a greater level of differentiation. These findings suggest that the organoids generated under suspension culture conditions are composed primarily of differentiated intestinal epithelial cells. These findings are consistent with prior reports suggesting that suspension culture systems may enhance differentiation at the expense of stem cell maintenance. Notably, Csukovich demonstrated that prolonged suspension culture of canine intestinal organoids resulted in polarity reversal, decreased stem cell marker expression, and reduced proliferative capacity, highlighting potential limitations of prolonged Matrigel-free culture systems [50]. Although polarity was not directly evaluated in this study, the reduced expression of LGR5 and increased MUC2 expression observed in our suspension-cultured duck organoids may reflect a similar trend toward differentiation and stem cell exhaustion.

WRN cell lines, which secrete high levels of Wnt3a, R-spondin 1, and Noggin, have been established through the culture of mouse fibroblasts as a replacement for ENR medium [51]. However, the supernatant produced by L-WRN cells is subject to batch effects, which may affect the reproducibility of the experimental results [17]. This issue highlights the importance of finding stable and suitable substitute components. CHIR99021, a glycogen synthase kinase-3β inhibitor, prevents the phosphorylation and degradation of β-catenin, thereby activating the Wnt signaling pathway and promoting the expression of stem cell markers [52]. Additionally, CHIR99021 significantly upregulated the expression of *SPDEF*, which plays a key role in goblet cell differentiation [53,54]. Moreover, studies have indicated that CHIR99021 can regulate the expression of *KLF4*, which indirectly participates in the regulation of goblet cell differentiation [55]. This mechanism provides theoretical support for the high expression of *MUC2* observed in the CL group in this study. Furthermore, BMP has been shown to limit the expansion of intestinal stem cells, thereby inhibiting the proliferation of the intestinal epithelium [56]. Thus, inhibiting the BMP signaling pathway is essential during organoid culture. LDN193189, by inhibiting the activity of BMP type I receptor kinase, exerts effects similar to those of Noggin. In mouse organoid culture, LDN193189 promotes the overall proliferation of intestinal epithelial cells, thereby facilitating intestinal organoid formation [29,57]. Using CHIR99021 and LDN193189 as substitutes for R-spondin 1 and Noggin, respectively, significantly increased the self-renewal capacity of intestinal stem cells and promoted the differentiation of Paneth cells, goblet cells, and enteroendocrine cells [29]. These findings are consistent with the results of this study, further validating the effectiveness of culture medium optimization.

Activin A, a member of the TGF-β superfamily, is widely present in animal tissues and organs and plays a role in promoting cell proliferation [58]. During the first three days of culture, activin A positively influences the survival and self-renewal capacity of cell colonies. However, in later growth stages, this molecule may have adverse effects [59]. Moreover, studies have shown that adding activin A on the third or fourth day to induce the formation of endoderm from pluripotent stem cells is a critical step in intestinal organoid culture [60]. The differences in the findings of this study may be attributed to species-specific variations. ZINC00881524, an inhibitor of Rho-associated protein kinase (ROCK), is involved in regulating cell proliferation, adhesion, and differentiation. ROCK pathway inhibition reportedly increases the differentiation and proliferation of bone marrow mesenchymal stem cells [61] and facilitates the recovery of pluripotent stem cells [62]. Furthermore, the suppression of ROCK signaling has been identified as essential for promoting the proliferation of rabbit intestinal organoids [33]. In contrast, in this study, the addition of ZINC00881524 accelerated organoid apoptosis compared with that in the CL medium. Nevertheless, the combined use of activin A and ZINC00881524 increased the expression of Paneth cells and enteroendocrine cells, suggesting a potential synergistic effect in promoting intestinal organoid differentiation.

TGF-β interacts with the Wnt signaling pathway and plays a crucial role in regulating intestinal epithelial cell proliferation, terminal differentiation, and migration, acting as a key signaling pathway in intestinal development [63,64]. In studies of mouse intestinal organoids, as the culture duration increased, the expression level of TGF-β significantly increased, accompanied by the upregulation of Smad3 and inhibitor of differentiation 1, which inhibited the proliferation and differentiation of intestinal epithelial cells. However, when TGF-β signaling was inhibited with SB431542, the regeneration and differentiation of mouse intestinal organoids were restored [65]. These findings differ from the observations in this study, potentially because SB431542 was added at the initial stage of culture, which inhibited the role of low levels of TGF-β in promoting intestinal epithelial cell proliferation and differentiation. In fact, moderate inhibition of TGF-β signaling appears to support the survival and proliferation of intestinal stem cells [66]. P38 MAPK, an important member of the MAPK signaling pathway, is involved in processes such as the cell cycle, differentiation, apoptosis, and senescence [67]. Studies suggest that the SB203580 inhibitor may suppress the differentiation of goblet cells and enteroendocrine cells [68]. However, when SB203580 was added to organoid culture media containing low concentrations of Wnt, Noggin, and R-spondin 1, the expression levels of intestinal stem cell and goblet cell markers significantly increased [69]. This observation aligns with the results of this study, suggesting that low concentrations or the absence of Wnt, Noggin, and R-spondin 1 may be key factors driving this phenomenon.

VA and RA promote the proliferation and migration of intestinal crypt stem cells, playing crucial roles in maintaining stem cell pluripotency [70,71]. Research has demonstrated that high concentrations of VA and RA can suppress the expression of *CHGA* and *MUC2*, thereby increasing stem cell activity by inhibiting organoid differentiation [20]. In addition, RA has been found to promote intestinal barrier function by increasing the expression of zonula occludens-1 and *MUC2*, further supporting its regulatory role in the gut [72]. This finding aligns with the findings of our study, where the addition of VA or RA resulted in a significant increase in *MUC2* expression levels. In this study, we further tested the effects of three different concentrations of VA or RA on organoid growth. We observed that as the concentration of VA or RA increased, the expression levels of *CHGA*, *MUC2*, *LYZ*, and *SI* gradually decreased. These results suggest that the inhibitory effects of VA or RA on intestinal cell differentiation may be negatively correlated with their concentration.

Existing chicken organoid studies have demonstrated their utility in investigating pathogen invasion routes such as Salmonella Typhimurium [73,74] and evaluating the effects of dietary additives on epithelial barrier function [73]. In ducks, intestinal health is particularly challenged by pathogens that directly target or interact with the gut epithelium. For example, Riemerella anatipestifer infection, a major bacterial disease in duck farming, induces strong intestinal and systemic immune responses [75]. In addition, Salmonella species are important zoonotic pathogens that can colonize the duck intestine and compromise food safety [76]. Viral pathogens such as duck plague virus (Duck herpesvirus-1) also enter through the digestive tract and cause intestinal lesions during early infection [77]. These disease contexts highlight the potential of duck intestinal organoids as in vitro platforms for investigating host–pathogen interactions at the epithelial interface. Beyond infectious diseases, the system may also be applied to assess the impact of feed additives, probiotics, and immunomodulators on intestinal homeostasis, thereby providing insights into nutritional and health management in duck production. Nevertheless, the present model also has several limitations. The relatively short culture period may restrict assessment of long-term stability and maturation. Although LGR5 immunostaining was performed, the resolution and imaging modality were insufficient for a comprehensive evaluation of apical–basal polarity, lumen formation, and the spatial organization of stem and differentiated cells within the organoids. Furthermore, the ROCK inhibitor ZINC00881524, unlike the widely used Y-27632, has rarely been applied in organoid research and lacks systematic validation. Its effects observed here suggest potential species-specific differences and reagent-dependent variability, which may influence the generalizability of the results. In addition, the relatively small sample size may also limit the extrapolation of the conclusions. These constraints indicate that further optimization, validation, and expansion will be necessary to fully unlock the potential of duck intestinal organoids.

## 5. Conclusions

In this study, we developed an initial duck intestinal organoid culture system by optimizing crypt isolation, culture method, and medium composition. Among the tested digestive solutions, EGTA was more effective than EDTA or collagenase for isolating duck intestinal crypts. Suspension culture improved organoid growth, survival, and differentiation compared to traditional embedding and air–liquid interface methods. A defined medium containing CHIR99021 and LDN193189 partially replaced conventional growth factors, supporting stem cell maintenance and promoting the differentiation of goblet, Paneth, and enteroendocrine cells. Although further optimization is required to achieve long-term expansion and full functional maturity, this system provides a reproducible platform that can serve as a basis for future research on intestinal development and function in ducks.

## Figures and Tables

**Table 1:** RT-qPCR primers in this study.

Gene	Sequences(5′-3′)	NCBI Number
*LGR5*	F: GCCTTTGTAGGCAACCCTTC R: AGGCACCATTCAAAGTCAGTG	NM_001315762.1
*CHGA*	F: ACTCCGAGGAGATGAACGGA R: CTTGGAGGACGCCTCTTCTG	NM_001164005.2
*MUC2*	F: GCTCCAGAGAGAAGGCAGAACC R: CTCAGGTGCACAGCGAACTC	XM 021082584.1
*LYZ*	F: TAACACGCAGGCTACAAAC R: TCCATCGCTGACAATCC	XM_005008880.5
*SI*	F: TTCCCAGACTTCTTACGC R: ACGCTGCTCACCTTCC	XM_038183707.1
*GAPDH*	F: ATCACAGCCACACAGAAGACG R: TGACTTTCCCCACAGCCTTA	NM_204305

## Data Availability

The original contributions presented in this study are included in the article. Further inquiries can be directed to the corresponding author.
